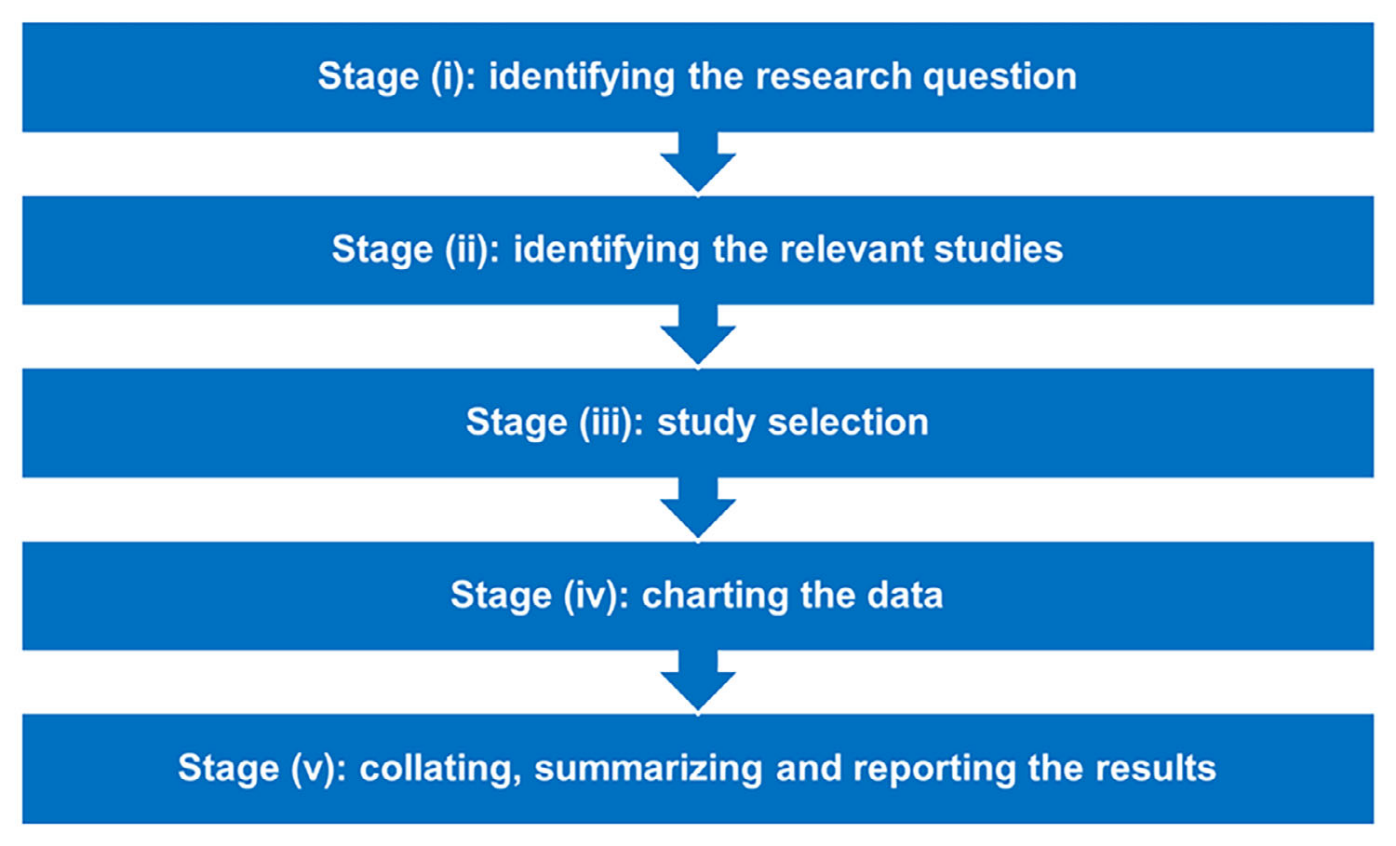# Age is not just a number to say 'it's early‐onset dementia' in workplace settings: A Scoping Review of Misinterpretation, Managerial Knowledge, and Organizational Responses

**DOI:** 10.1002/alz70858_107429

**Published:** 2025-12-25

**Authors:** Shruti Jindal, Mohammad Hamiduzzaman

**Affiliations:** ^1^ Jamia Millia Islamia, New Delhi, New Delhi, India; ^2^ University of Sydney, Camperdown, NSW, Australia

## Abstract

**Background:**

This scoping review explores the misinterpretation of symptoms associated with early‐onset dementia versus normal aging within workplace settings. Distinctions between these symptoms are critical for appropriate management and support, yet misconceptions persist, particularly among managers who may not have specialized knowledge of dementia.

**Method:**

Employing Arksey and O'Malley's scoping review framework and adhering to PRISMA‐P guidelines, we conducted a systematic search of literature published since 2020. Our databases included PubMed and PsycINFO, utilizing a comprehensive array of search terms and boolean operators. The focus was on studies that specifically address early‐onset dementia symptoms and their misinterpretation in workplace environments. The selection criteria were designed to include only those studies that directly addressed these phenomena, thus maintaining a tight scope to ensure relevance and specificity to our research objectives.

**Result:**

The review uncovered a substantial lack of understanding among managers regarding the unique needs and challenges faced by employees with early‐onset dementia. Specific gaps identified include the absence of tailored training programs that equip managers to recognize and effectively support dementia symptoms in the workplace. Additionally, our findings pointed to insufficient workplace policies to facilitate necessary adjustments, such as flexible scheduling and task modification. It also became evident that organizational support mechanisms are largely underdeveloped, lacking specialized occupational health services that are pivotal for effective dementia management in professional settings.

**Conclusion:**

This review underscores the urgent need for organizational change to enhance the recognition and management of early‐onset dementia within the workforce, highlighting deficiencies in current workplace practices and managerial knowledge.